# PTBP3 contributes to colorectal cancer growth and metastasis via translational activation of HIF-1α

**DOI:** 10.1186/s13046-019-1312-y

**Published:** 2019-07-10

**Authors:** Pingfu Hou, Fang Chen, Hongmei Yong, Tian Lin, Jingjing Li, Yu Pan, Tao Jiang, Minle Li, Yansu Chen, Jun Song, Junnian Zheng, Jin Bai

**Affiliations:** 1 Cancer Institute, Xuzhou Medical University, 84 West Huaihai Road, Xuzhou, 221002 Jiangsu Province China; 2 Center of Clinical Oncology, Affiliated Hospital of Xuzhou Medical University, Xuzhou, China; 3 School of Medical Imaging, Xuzhou Medical University, Xuzhou, Jiangsu China; 4 School of Public Health, Xuzhou Medical University, Xuzhou, Jiangsu China; 5 Department of General Surgery, Affiliated Hospital of Xuzhou Medical University, Xuzhou, Jiangsu China; 6 Department of Medical Oncology, Huai’an Hospital to Xuzhou Medical University, Huai’an, Jiangsu Province China

**Keywords:** PTBP3, Colorectal cancer, HIF-1α, Protein translation, Tumor metastasis

## Abstract

**Background:**

Colorectal cancer (CRC) remains the fourth most common cause of cancer-related mortality worldwide. We aimed to identify key molecules and signalling pathways mediating CRC growth and metastasis. Polypyrimidine tract-binding protein 3 (PTBP3) is a member of PTB family. A prooncogenic role for PTBP3 has also been discovered in several kinds of tumors. However, the expression and biological functions of the PTBP3 are still unknown in CRC.

**Methods:**

We analysed the expression levels of PTBP3 using tissue microarray containing 568 CRC tissues and corresponding non-tumor adjacent tissues. The correlations between the PTBP3 expression level and clinicopathological features were evaluated using the chi-square test. The functional characterization for the role and molecular mechanism of PTBP3 in CRC was investigated through a series of in vitro and in vivo experiments.

**Results:**

We showed that PTBP3 expression was increased in human CRC, and high PTBP3 expression was correlated with poor five-year overall survival and disease-free survival. Moreover, PTBP3 promoted tumor cell proliferation, migration and invasion in vitro and tumor growth and metastasis in vivo. PTBP3 enhanced HIF-1α protein expression by directly binding to the 5′UTR HIF-1α mRNA and activated translation of HIF-1α. Furthermore, HIF-1α was responsible for PTBP3-induced cell migration and invasion.

**Conclusions:**

PTBP3 appears to be a novel oncogene of CRC through binding to the IRES region of HIF-1α mRNA, which regulates HIF-1α translation. PTBP3 can serve as a promising predictive biomarker for recurrence and prognosis in patients with CRC.

**Electronic supplementary material:**

The online version of this article (10.1186/s13046-019-1312-y) contains supplementary material, which is available to authorized users.

## Background

Colorectal cancer (CRC) is a major lethal malignancy worldwide, and the incidence rates of CRC increased rapidly years recently in China [1]. Patients with distant metastasis suffer from poor prognosis, and distant metastasis has become one of the leading causes of death in cancer patients. Approximately 50% of CRC patients die from developing distant metastasis [2]. Therefore, discovering novel metastasis promoters and suppressors is essential.

RNA-binding proteins (RBPs) are increasingly identified as post-transcriptional drivers of cancer progression. Polypyrimidine tract-binding protein 3 (PTBP3) is also known as regulator of differentiation 1, which was first discovered in 1999 as an RNA binding protein, highly expressed in hematopoietic cells [3]. PTBP3 is a member of PTB family. The PTB family has three paralogs, namely, PTBP1, PTBP2 and PTBP3. The functions and developmental roles of PTBP1 and PTBP2 have been well characterized. PTBP1 and PTBP2 can interact with regulatory RNAs to regulate mRNA splicing, stability, localization and translation [4]. By contrast, little information about cellular functions or physiological roles of PTBP3 is known. Recently, we have discovered that PTBP3 was located both in nuclear and cytoplasm; and that PTBP3 promoted breast cancer EMT and metastasis by repressing ZEB1 mRNA degradation [5]. A prooncogenic role for PTBP3 has also been discovered in governing gastric cancer cell cycle and growth [6]. An improved understanding of the regulatory mechanism about PTBP3 in regulating cancer metastasis can provide critical information on how to block related processes in cancer progression.

Hypoxia is a common phenomenon in solid malignancies, including CRC. The hypoxia-inducible transcription factors 1α (HIF-1α) and 2α are broadly expressed in many human cancers, and expression of these proteins frequently correlate with poor patient prognosis [7]. The roles of HIF-1α have been well documented in cancer progression and implicated in CRC metastasis [8, 9]. HIF-1α protein expression level can be regulated at post-translational levels, such as hydroxylation, acetylation, ubiquitin and phosphorylated modification [10]. Recently, YBX1 activates HIF-1α at the translation level by binding to the IRES sequences of the HIF-1α mRNA 5’UTR region to promote sarcoma metastasis [11]. However, the translational mechanism of HIF-1α is still poorly understood in cancer progression, even though the translational component contributes to approximately 40–50% of HIF-1α total proteins under hypoxia [12].

In the current study, we investigated the expression of PTBP3 in clinical CRC samples of patients and its potential role and molecular mechanism in regulating CRC malignant features. We found that PTBP3 was markedly upregulated in CRC tissues of patients compared with the adjacent normal colon tissues and was positively correlated with poor five-year survival of CRC patients. Enhanced PTBP3 expression promoted colon cancer cell proliferation, migration, and invasion in cultured cells, and also increased tumor growth and metastasis in animal model. Importantly, we found that PTBP3 regulated HIF-1α protein expression by enhancing HIF-1α translation. We also showed that blocking HIF-1α pathway using HIF-1α inhibitor 2-Methoxyestradiol (2-MeOE2) significantly reduced tumor growth formed by PTBP3 overexpression (OE) cell. These data indicated that PTBP3 can be a potential biomarker and therapeutic target for CRC.

## Methods

### Patients and sample collection

The tissue microarrays (TMAs) slides included examination of 568 pairs of tissue specimens including 568 CRC tissues and the matched corresponding adjacent normal colorectal tissues from CRC patient cohorts that were enrolled at Affiliated Hospital of Xuzhou Medical University from 2010 to 2015 in China (As described in our previous work [13]). The average age of the patients was 61.7 years old and approximately 57.6% were male and 42.4% were female. For clinicopathologic factors, 61.4% patients were at tumor stage I and II, 38.9% were at tumor stage III and IV. Survival time was calculated based on the date of surgery to the date of death or to the last follow-up. The patient studies were conducted in accordance with Declaration of Helsinki. The use of these specimens and data for research purposes were approved by the Ethics Committee of the Hospital.

### Cell lines and cell culture conditions

The CRC cell lines were obtained from the cell bank of Chinese academy of sciences. HCT116, SW480, SW620, DLD1 and embryonic kidney 293 T cells were cultured in DMEM Medium supplemented with 10% fetal bovine serum, 100 U/ml penicillin, 100 μg/ml streptomycin, and incubated in a 37 °C humidified incubator with 5% CO_2_. One percent O_2_ was generated by flushing a 94% N_2_/5% CO_2_ mixture into the incubator.

### Western blot and antibodies

Western blot was performed as previously described [5]. Antibody against PTBP3 (sc-100845, Santa Cruz, Dallas, TX, USA), GAPDH (sc-32233, Santa Cruz, Dallas, TX, USA), VEGF (19003–1-AP, Proteintech Group, Wuhan, China), HIF-1α (ab51608, Abcam), YBX-1 (20339–1-AP, Proteintech Group, Wuhan, China), HNRNPM (26897–1-AP, Proteintech Group, Wuhan, China), IGF2BP1 (22803–1-AP, Proteintech Group, Wuhan, China), UPF1 (23379–1-AP, Proteintech Group, Wuhan, China) were used for Western blot assays. Each western blot was performed at least three times.

### Establishment of stable cell lines

PTBP3 overexpression and knockdown vector was cloned as described previously [5]. In brief, PTBP3 cDNA was cloned to the pCDH1-CMV-MSC-EF1-GFP-Puro vector and the PTBP3 shRNAs or scrambled sequences were cloned to the pLKO.1 vector. To obtain PTBP3 OE and KD lentiviruses, PTBP3 OE/KD or empty vector was transfected into 293 T together with the packing plasmids (psPAX and pMD2G) for producing viral particles using Lipofectamine 2000 (Invitrogen). And then, Stable cell lines overexpressing or lacking PTBP3 were generated by infecting with lentivirus and selected with 2 mg/mL puromycin for about 2 weeks. The PTBP3 shRNA sequences were described as bellow: shPTBP3#1-for: CCGGACCAGGAAATTCTGTTCTACTCTCGAGAGTAGAACAGAATTTCCTGGTTTTTTG, shPTBP3#2-for: CCGGCAGAGACTTCACTCGCTTAGACTCGAGTCTAAGCGAGTGAAGTCTCTGTTTTTG.

### Immunohistochemistry (IHC) and statistical analysis

IHC assays were performed as previously described [13]. For primary antibody incubation, anti-PTBP3 (Santa Cruz Biotechnology) antibody was applied at 1:100 dilution, anti-HIF-1α (MA1–16511, Thermo Fisher) antibody at 1:100 dilution, anti-CD31 (11265–1-AP, Proteintech, China) antibody at 1:50 dilution, anti-VEGF (Abcam) antibody at 1:100 dilution.

### Immunofluorescence

Cells were seeded on glass coverslips in 24-well plates for about 24 h, then cells were fixed with 4% formaldehyde, permeabilized with 0.2% Triton X-100, blocked with 5% bovine serum albumin in PBS. After incubation with primary antibody (anti-PTBP3, 1:100 dilution) at room temperature for 2 h, cells were washed three times with PBST and followed by incubation with FITC conjugated secondary antibodies for 1 h, and then stained with 2 μg/ml Hoechst. Finally, images were taken under a confocal microscope.

### RNA extract, reverse transcription-PCR and qRT-PCR

RNA was extracted using TRIzol (Invitrogen) and cDNA was synthesized using the HiScript 1st Strand cDNA Synthesis Kit (Vazyme Biotech, Nanjing, China). Real-time PCR was carried out on ABI-7500 using UltraSYBR One Step RT-qPCR Kit (CWBIO, Beijing, China). The primers using for quantitative RT-PCR analysis were listed below:

- GAPDH-For: AAGGTCGGAGTCAACGGATTTG,
- GAPDH-Rev: CCATGGGTGGAATCATATTGGAA;
- PTBP3-For: ACAGCTAATGGGAATGACAGCA,
- PTBP3-Rev: CTGGCTTCGAAGGTGAGGAG;
- HIF1A-For CCCAATGTCGGAGTTTGGAA,
- HIF1A-Rev GTGGCAACTGATGAGCAAGC;
- U6-For: CTCGCTTCGGCAGCACA,
- U6-Rev: AACGCTTCACGAATTTGCGT.
- HIF-5′UTR-For: AGTCTCACGAGGGGTTTCCC,
- HIF-5′UTR-Rev: CTCCACACGCGGAGAAGAG;

### Luciferase reporter assay

HIF-1A 5′UTR cDNA sequence or hypoxia response element [14] of VEGF were synthesized (Genewiz Company, Suzhou, China.) and cloned to the pGL4.20 vector (Promega) at the upstream of the luciferase sequence. The pGL4.20-HIF1A-5′UTR-LUC or pGL-4.20-7 × HRE and renilla vectors were transiently transfected into ± PTBP3 OE cells using Lipofectamine 2000. At 48 h post-transfection, cells were lysed and analyzed for firefly luciferase (Fluc) activity and renilla luciferase (Rluc) using the Dual Luciferase Reporter Assay System (Promega). The Rluc activity was used for normalization.

### Cell migration and invasion assays

Cells were starved in serum-free media for 24 h then added to the top chamber of 24-well transwell chambers plates (8.0 μm, Corning, NY, USA.). Specially, for the invasion assay, cells were seeded into the top chamber coated with Matrigel (BD Biosciences). Complete medium was added to the bottom chambers to stimulate migration or invasion. After incubation for 24–48 h, the cells those adhered to the lower surface of the membrane were stained with 0.1% Crystal Violet, and then the percentages of migrated or invasion cells were calculated. Five randomly selected fields per filter were counted.

### Cellular proliferation and colony formation assays

CCK-8 assay was applied to measure the cell proliferation according to the Cell Counting Kit-8 manufacturer’s protocol (Dojindo). Colony formation assay was performed as described previously [5].

### RNA immunoprecipitation

The RIP experiment was carried out with the EZ-Magna RIP Kit (Millipore) according to the manufacturer’s protocol using 5 μg of anti-Flag antibody (Sigma). The primers using for quantitative RT-PCR analysis was listed above.

### In vitro transcription and RNA pull-down

The HIF-1A 5′-UTR sequence was cloned from the pGL-4.20-HIF-1A-5′-UTR plasmid by PCR using primers containing a T7 promoter. Then, 500 ng HIF-1α-5′-UTR DNA was used as a temple to synthesize RNA using T7 RNA polymerase Transcription system (Promega) and Biotin-16-UTP (Roche), according to the manufacturer’s instructions. Two micrograms of biotinylated RNA was used to performed the RNA pull-down assay following the protocol as previously described [15]. In Brief, Two micrograms of biotinylated RNA was heated to 90 °C for 2 min, put on ice for 2 min, supplied with RNA structure buffer (10 mM Tris pH 7, 0.1 M KCl, 10 mM MgCl2), and then shifted to room temperature (RT) for 20 min to allow proper secondary structure formation. Then, folded RNA was then mixed with 1 mg of cell extract in RIP buffer ((150 mM KCl, 25 mM Tris pH 7.4, 0.5 mM DTT, 0.5% NP40, 1 mM PMSF, RNasin (NEB) and protease Inhibitor (Roche)) and incubated at RT for 1 h. Next, 60 microliters washed Streptavidin agarose beads (Thermo fisher) were added to each binding reaction and further incubated at RT for 1 h. At last, beads were washed briefly five times and boiled in SDS loading buffer, and the retrieved protein was detected by standard western blot. For RNA pull-down assay using recombinant proteins, five micrograms of GST-PTBP3 fusion protein (Ag5172, Proteintech Group, Wuhan, China) was used for one assay, the assay was performed following the protocol as described before [15].

### Animal work

The female BALB/c nude mice (6–8 weeks old) were purchased from Beijing Vital River Laboratory Animal Technology Co., Ltd. (Beijing, China). All animal experiments were approved by the Animal Care and Use Committee at Xuzhou Medical University. Animal experiments were performed as described previously. [5] Specially, for the mice treating with 2-MeOE2 (S1233, Selleck), the mice were treated with 2-MeOE2 (50 mg/kg) by means of intragastric administration for 7 days after 5 days past tumor cells formed xenografts. The tumor volume was calculated using the formula V = a × (b × b)/2, where a is the largest and b is the smallest diameter.

### Bioinformatics of gene expression database

To determine the correlation of PTBP3 mRNA expression with HIF-1A target genes. We analyzed the datasets generated with Affymetrix HGU133 Plus 2.0 microarrays from published studies. Data are deposited at the Gene Expression Omnibus (GSE40967). GSE40967 contains 566 CRC tissues. The average of the probes for the analyzed genes was used in all analysis.

### Statistical analysis

Statistical analyses were carried out using SPSS 20.0 software (SPSS Inc., Chicago, IL, USA) and GraphPad Prism 7. The association between PTBP3 staining and the clinicopathologic parameters of the CRC patients were evaluated by a Chi-square test. The Kaplan–Meier method and log-rank test were used to evaluate the correlation between PTBP3 expression and CRC patient survival. The unpaired t test was used to determine the statistical significance of differences between groups. Data were presented as mean ± SD. *p* < 0.05 was considered statistically significant.

## Results

### PTBP3 was overexpressed in patients with CRC and was associated with poor outcome

**Fig. 1:**
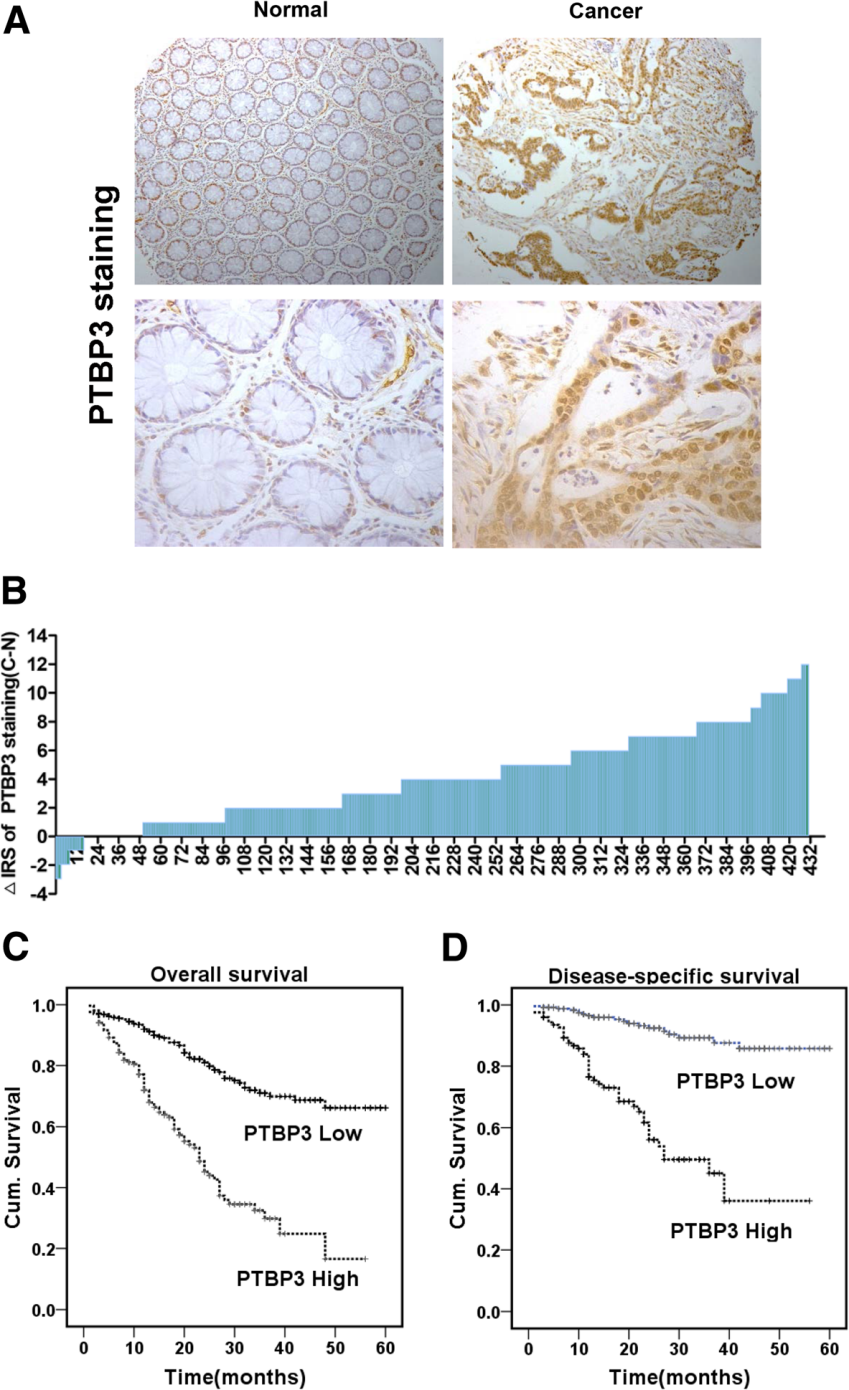
PTBP3 expression in colorectal tumors predicts clinical outcome. **a** Representative immunohistochemistry images of PTBP3 protein expression in paired adjacent non-cancerous tissue and colorectal carcinoma tissues in CRC patients. **b** Staining intensities of PTBP3 in colorectal carcinoma tissues compared with paired adjacent non-cancerous tissue. N, paired adjacent non-cancerous tissues. C, colorectal carcinoma tissues (*p* < 0.001). **c-d,** Kaplan–Meier survival curves depicting overall survival (*n* = 479, *p* < 0.001) or disease-specific survival of patients with CRC (*n* = 372, *p* < 0.001) stratified by PTBP3 protein expression levels in CRC tissues

To investigate the role in the development of CRC, we assessed PTBP3 protein expression by IHC in CRC patients using tissue microarray (TMA) slides (Fig. 1a). Results showed that the PTBP3 expression was dramatically upregulated in cancerous tissue compared with adjacent non-cancerous tissue in CRC patients (Fig. 1b) (*p* < 0.001). The correlation between PTBP3 expression and clinicopathologic characteristics in CRC tissue samples was analyzed (Table 1). Compared with histology grade I and II, the expression of PTBP3 was dramatically increased in stage III and IV (*p* < 0.001). In addition, high PTBP3 expression was positively correlated with the depth of invasion (*p* = 0.032).

**Table 1:** Relationship between PTBP3 expression and clinicopathological features of CRC patients

Variables	PTBP3 expression (*n* = 479 cases)		
	Low	High	*P* ^a^
All patients	311	168	
Age (years)			0.081
≤ 60	139	61	
> 60	172	107	
Gender			0.495
Males	189	96	
Females	122	72	
Depth of invasion			0.032*
T1/T2	72	25	
T3/T4	239	143	
Lymph node metastasis			0.074
N0	206	97	
N1/N2/N3	105	71	
Distant metastasis			0.347
M0	300	159	
M1	11	9	
TNM stage			< 0.0001***
I/II	168	47	
III/ IV	143	121	
Tumor diameter			0.250
<5 cm	162	97	
≥ 5 cm	149	71	
Differentiation			0.437
Poor	47	30	
Moderate/high	264	138	

^a^Two -sided Fisher’s exact tests

*p < 0.05, ***p < 0.001

Kaplan–Meier survival analysis was performed to evaluate the impact of PTBP3 expression on the overall (OS) and disease-free survivals (DFS) in patients with CRC. The results revealed that high PTBP3 levels were correlated with poor OS (*p* < 0.001, log-rank test, Fig. 1c) and DFS (*p* < 0.001, log-rank test, Fig. 1d). Furthermore, we revealed high PTBP3 expressions were not correlated with poor OS in histology grade I and II (Additional file 3: Figure S1A), but correlated with poor OS in histology grade III and IV (*p* < 0.001, Additional file 3: Figure S1B), in stage pT1/T2 (*p* < 0.001, Additional file 3: Figure S1C), in stage pT3/T4 (*p* < 0.001, Additional file 3: Figure S1D), in stage pN0 (*p* < 0.001, Additional file 3: Figure S1E), and in stage pN1/N2/N3 (*p* < 0.001, Additional file 3: Figure S1F).

To further examine whether PTBP3 expression was an independent prognostic factor for CRC, we used univariate and multivariate Cox regression analysis models to confirm the prognostic value of PTBP3 expression in CRC. The univariate Cox regression analysis suggested that PTBP3 expression was a significant prognostic factor for OS and DFS of CRC patients (Additional file 1: Table S1). In multivariate Cox regression analysis, we also found that PTBP3 expression was also an independent prognostic marker for both OS (HR 2.484, 95%CI 1.761 to 3.502, *p* < 0.001) and DFS (HR 4.830, 95%CI 2.763 to 8.445, *p* < 0.001) in patients with CRC (Additional file 2: Table S2). Therefore, we further assessed the biological functions and molecular mechanisms of PTBP3 in CRC development.

### PTBP3 was essential for colon cancer cell growth in vitro and in vivo

**Fig. 2:**
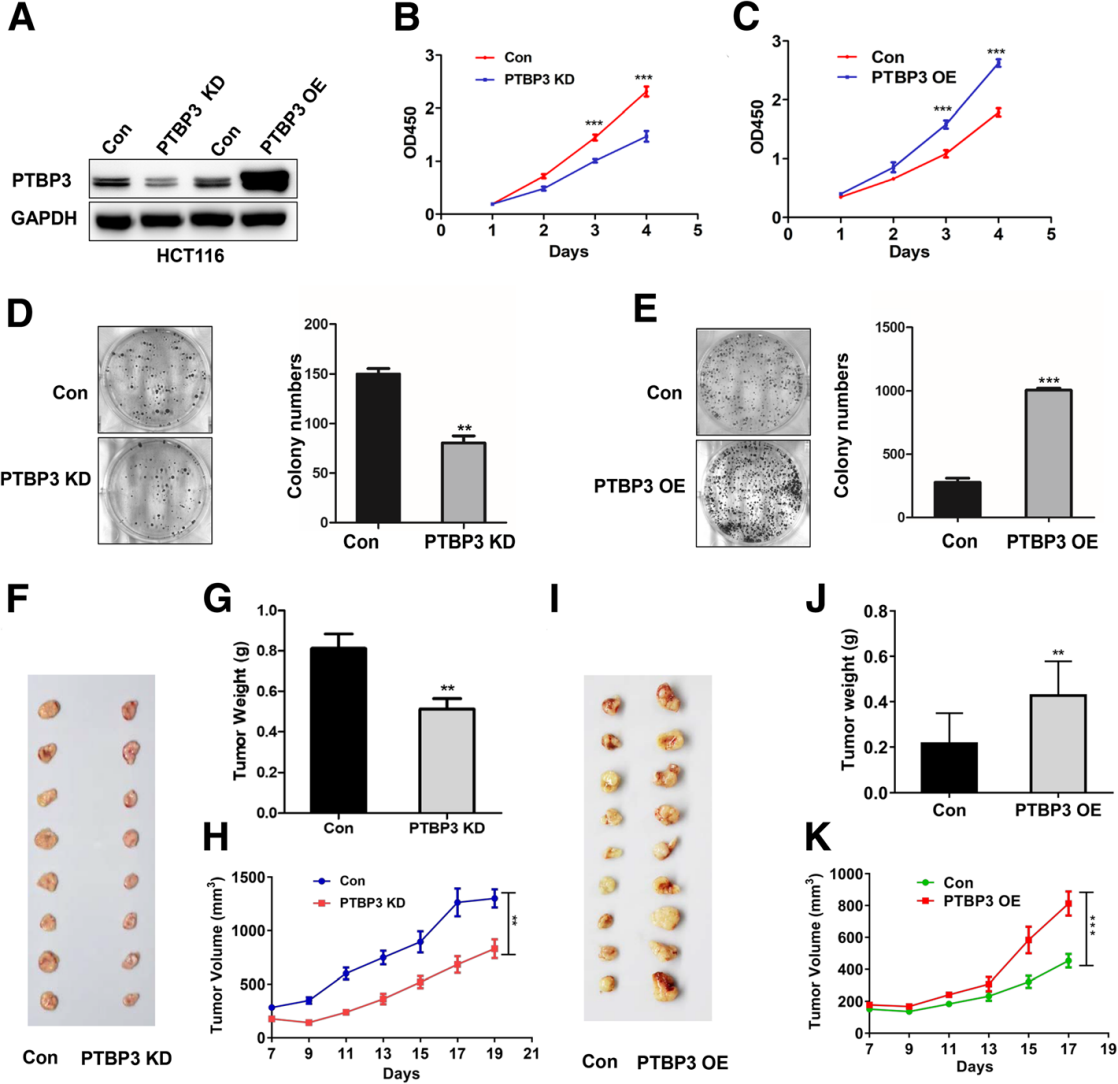
Effects of PTBP3 on tumor growth in cultured cells and an animal model of colorectal cancer. **a** Detection of PTBP3 protein level in PTBP3 KD and OE HCT116 stable cell lines. PTBP3 KD used the mix pool of two shRNAs(#1 and #2) of PTBP3. GAPDH was used as a loading control. **b-c** Effect of PTBP3 KD or OE on HCT116 cell proliferation as assessed by Cell Counting Kit-8 (CCK8) assays. **d-e** Colony formation assays for PTBP3 KD or OE in HCT116 cell lines. The number of colonies with > 50 cells was counted after 10-day incubation. **f-h** Effect of PTBP3 KD in HCT116 cells on the xenograft model was assessed by evaluating tumor volume and tumor weight. 5 × 10^6^ HCT116 PTBP3 Con/KD cells and Matrigel (Corning; 1:1 ratio) were subcutaneously injected into the abdominal flanks of each mice, respectively. **i-k** Effect of PTBP3 OE in HCT116 cells on the xenograft model was assessed by evaluating tumor volume and weight. 2 × 10^6^ HCT116 PTBP3 Con/OE cells and Matrigel (Corning; 1:1 ratio) were subcutaneously injected into the abdominal flanks of each mice, respectively. A pool of two independent shRNAs (#1 and #2) was used for PTBP3 KD. Statistical analysis was performed using unpaired t-tests. All statistical tests were two-sided. **p < 0.01, ***p < 0.001

To determine whether PTBP3 regulates cell viability in colon cancer cells, we measured cell viabilities using cell counting kit-8 (CCK8) in HCT116 cells with PTBP3 stable knockdown (KD) and overexpression (OE) (Fig. 2a). The CCK8 assays showed that PTBP3 KD inhibited HCT116 cell proliferation (Fig. 2b). By contrary, HCT116 cell with PTBP3 OE exhibited significantly higher cell proliferation rates (Fig. 2c). In addition, colony formation assay also showed that PTBP3 expression was positively correlated with cell growth (Fig. 2d-e). These results suggested that PTBP3 was an essential factor for cancer cell growth in vitro. The findings were consistent with previous studies about PTBP3 promoting cell growth in breast cancer and gastric cancer [5, 16].

To confirm effects on tumor growth in vivo, we performed xenograft model using nude mice to assess the effects of PTBP3 KD/OE on colon cancer cell growth. Consistent with the in vitro assays, mice with xenografts of PTBP3 KD tumor cells formed smaller tumors both in tumor size and weight in vivo compared with the control group (Fig. 2f-h). PTBP3 OE promoted tumor growth in vivo (Fig. 2i-k). In conclusion, these results clearly demonstrated that high PTBP3 expression enhanced tumor growth in vitro and in vivo.

### PTBP3 increased colon cancer cell migration in vitro and metastasis in vivo

**Fig. 3:**
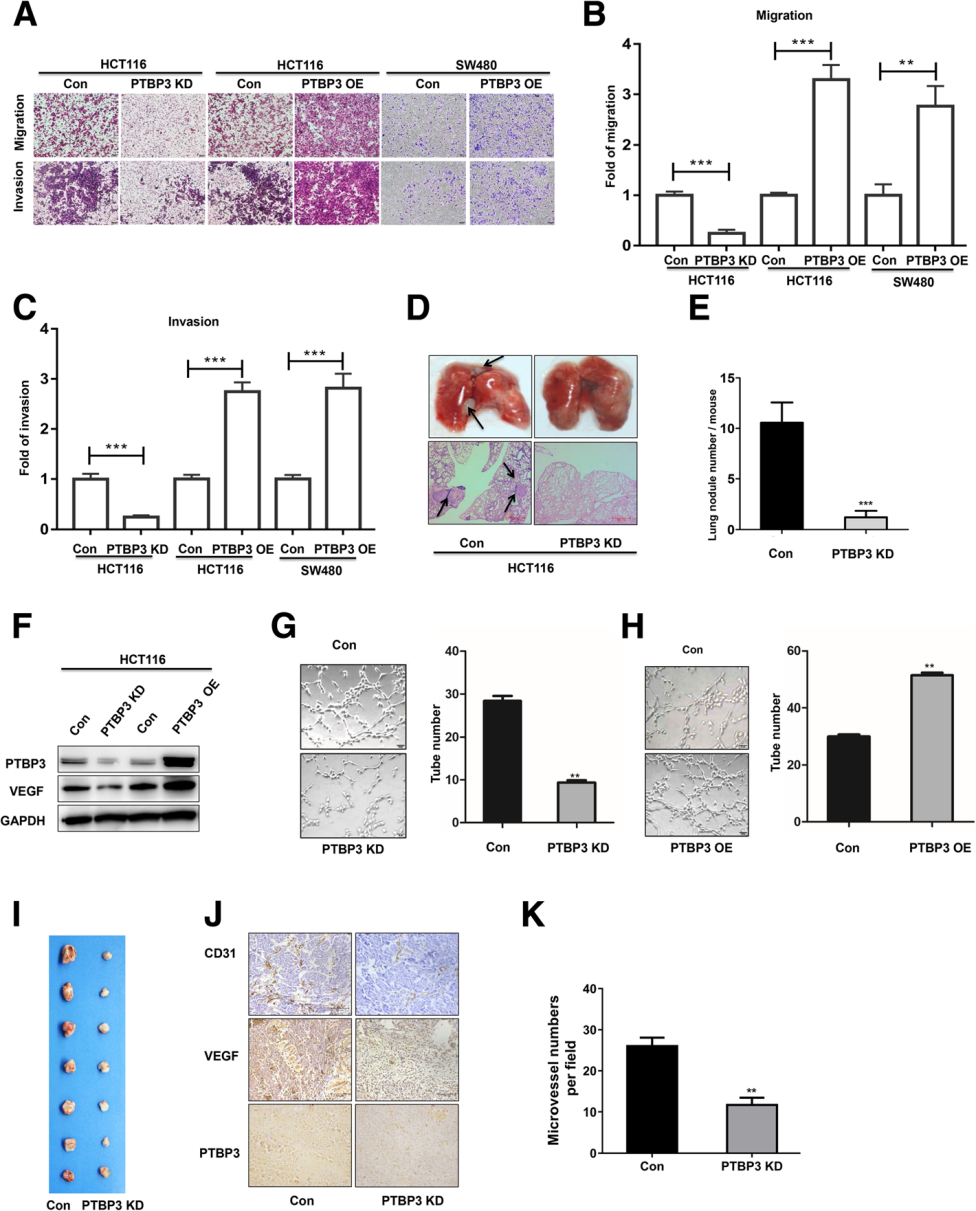
PTBP3 increases colon cancer cells motility and angiogenesis in vitro and in vivo. **a** Cell migration and invasion of HCT116 cells ± PTBP3 KD/OE, and SW480 cells ± PTBP3 OE measured as percent cells migrating to chambers. **b** Relative migration fold changes in HCT116 cells ± PTBP3 KD/OE, and SW480 cells ± PTBP3 OE. **c** Relative invasion fold changes in HCT116 cells ± PTBP3 KD/OE, and SW480 cells ± PTBP3 OE. **d** HCT116 cells ± PTBP3 KD were injected via the lateral tail veins, respectively. Representative lung images at week 5; corresponding hematoxylin/eosin–stained lung sections are shown. Arrows, lung metastasis foci. **e** Lung nodules at week 5 were analyzed as the numbers of nodules per mouse. *n* = 8. **f** Detection of VEGF protein expression using Western blot in HCT116 cells ± PTBP3 KD/OE. GAPDH was used as a loading control. **g-h** PTBP3 in CRC cells positively regulated tube formation. Numbers of complete tubular structures formed by HUVECs were counted for ± PTBP3 KD/OE in HCT116 cells. Data are presented as the means ± SD for experiments in triplicate. **i** Photographs of matrigel plugs with HCT116 cells ± PTBP3 KD excised from mice after 7 days of growth in vivo. **j** IHC detection of PTBP3, VEGF and CD31 in xenograft tumors formed by HCT116 cells ± PTBP3 KD after 7 days of growth in vivo. Scale bar: 100 μm. **k** Quantification of CD31 positive microvessel density in tumor xenografts formed by HCT116 cells ± PTBP3 KD. A pool of two independent shRNAs (#1 and #2) was used for PTBP3 KD. Statistical analysis was conducted using a two-tailed student’s t test, Data are presented as the means ± SD for experiments in triplicate. *p < 0.05, **p < 0.01,***p < 0.001

To investigate whether PTBP3 contributes to aggressiveness of colon cancer, we first tested the effects of PTBP3 on cell migration and invasion in vitro, two critical features of the metastatic phenotype [17]. Migration assays showed that PTBP3 KD and OE inhibited and increased the migration ability (Fig. 3a-b), respectively. Invasion assays also showed that PTBP3 KD and OE inhibited and increased the invasive ability (Fig. 3a and c), respectively. To detect the role of PTBP3 in colon cancer cells lung metastasis in vivo, we used tail vein injection mouse model. In brief, 1 × 10^6^ HCT116 Control/KD cells were injected via lateral tail vein to 7-week-old nude mice, and the lung metastasis status were analyzed 5 weeks later. PTBP3 KD cells formed fewer metastatic foci in lung compared with the control group (Fig. 3d), and PTBP3 KD also significantly reduced the number of lung metastases per mouse (Fig. 3e). These results provided strong evidence that PTBP3 promoted CRC aggressiveness by increasing cell migration, invasion and metastasis.

### PTBP3 promoted colon cancer cell angiogenesis

Given that our results have showed that PTBP3 was an essential factor for tumor growth and metastasis, and angiogenesis is widely believed to play an important role in tumor growth and metastasis [18]. We further studied the function of PTBP3 in CRC angiogenesis. We detected the vascular endothelial growth factor (VEGF) expression in PTBP3 stable KD or OE HCT116 cell lines, which showed the VEGF expression was decreased or increased, corresponding to PTBP3 depletion or OE, respectively (Fig. 3f). Furthermore, the conditioned medium was collected to perform the tube formation assays in vitro. The tube formation assays showed that the number of complete tubular structures formed by HUVECs significantly decreased and increased in the conditioned medium collected from PTBP3 KD and OE cells compared with the corresponding controls, respectively (Fig. 3g-h).

To investigate the suppressive role of PTBP3 in angiogenesis, we performed the in vivo Matrigel plug. The PTBP3 KD or control HCT116 cells mixing with Matrigel were injected subcutaneously into the nude mice to form neovessels, respectively. The visual examination revealed PTBP3 KD cells had reduced neovessels compared with the control cells (Fig. 3i). The IHC assays showed tumors formed by PTBP3 KD cells expressed lower angiogenesis makers CD31 (Fig. 3j), and formed fewer CD31 vessels compared with the control group (Fig. 3k). These data indicated that PTBP3 conferred angiogenesis capacity to colon cancer cells.

### PTBP3 increased HIF-1α protein levels in colon cancer cells

**Fig. 4:**
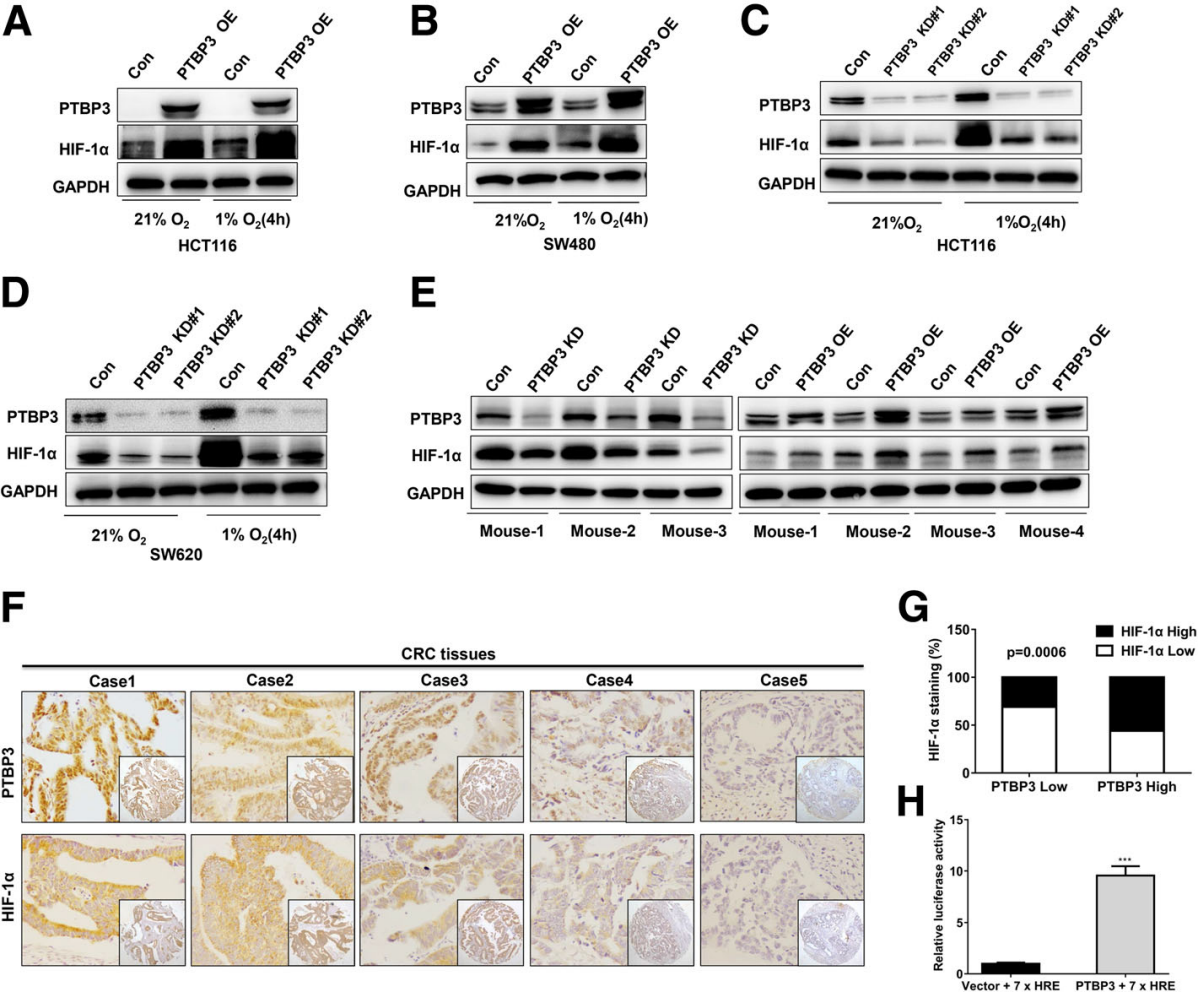
PTBP3 increases HIF-1α protein levels in colon cancer cells. **a-b** Western blot of HIF-1α expression in HCT116 and SW480 cells ± PTBP3 OE grown under normoxia or hypoxia (1% O_2_) for 4 h. GAPDH was used as a loading control. **c-d,** Western blot of HIF-1α expression in HCT116 and SW480 cells ± shRNA PTBP3 KD grown under normoxia or hypoxia (1% O_2_) for 4 h. GAPDH was used as a loading control. **e** Western blot of HIF-1α expression in tumor xenografts formed by HCT116 cells ± PTBP3 KD/OE. GAPDH was used as a loading control. **f** Representative examples of IHC-based correlation between PTBP3 and HIF-1α expression in colorectal tumor sections from TMAs. **g** Fisher′s exact test analysis of the correlation of PTBP3 and HIF-1α expression in CRC TMAs. **h** Relative HRE-Luc activity in HCT116 cells ± PTBP3 OE was detected by the Dual Luciferase Reporter Assay System. The Rluc activity was used for normalization. Data are presented as the means ± SD for experiments in triplicate. ***p < 0.001

Hypoxia is a common phenomenon in colon cancers and involves in tumor angiogenesis, growth and metastasis [19]. VEGF is a direct target of HIF-1α and plays an essential role in angiogenesis and tumor metastasis [20–22]. We have showed that PTBP3 can promote tumor growth, metastasis and angiogenesis, as well as, regulated VEGF expression, which is a direct target of HIF-1A gene. We therefore wondered whether PTBP3 can regulate the expression of HIF-1α protein. To determine whether HIF-1α protein was regulated by PTBP3 in colon cancer, we detected the expression of HIF-1α in PTBP3 OE and KD colon cancer cells. Our data showed that HIF-1α protein expression was significantly increased under normoxia and 1% O_2_ in PTBP3 OE colon cancer cell HCT116 and SW480 (Fig. 4a-b). PTBP3 KD markedly blocked HIF-1α protein levels under normoxia and 1% O_2_ in HCT116 and SW620 (Fig. 4c-d). These results suggested that PTBP3 can regulate HIF-1α expression in both normoxia and hypoxia.

We analyzed the expression of HIF-1α in xenografts formed by PTBP3 KD and OE HCT116 cells and found that the HIF-1α protein levels were markedly reduced in PTBP3 KD tumors and significantly increased in PTBP3 OE tumors in vivo (Fig. 4e). Finally, we immunostained colon cancer TMAs for PTBP3 and HIF-1α to assess whether PTBP3 regulated HIF1a in human colon cancers, The results showed that PTBP3 expression strongly correlated with HIF-1α IHC scores in colon cancer tissues (Fig. 4f-g). To confirm that PTBP3-induced HIF-1α is biologically active, we monitored activity of a hypoxia response element (HRE) reporter linked to luciferase in cells overexpressing PTBP3. This revealed the HRE activity was much higher in PTBP3 OE cells (Fig. 4h), suggesting that PTBP3 could active the HIF-1α signalling pathway. These results provide compelling evidence that PTBP3 positively regulates HIF1a protein expression in human colon cancers.

### PTBP3 directly binds to HIF-1α mRNA to activate its translation

**Fig. 5:**
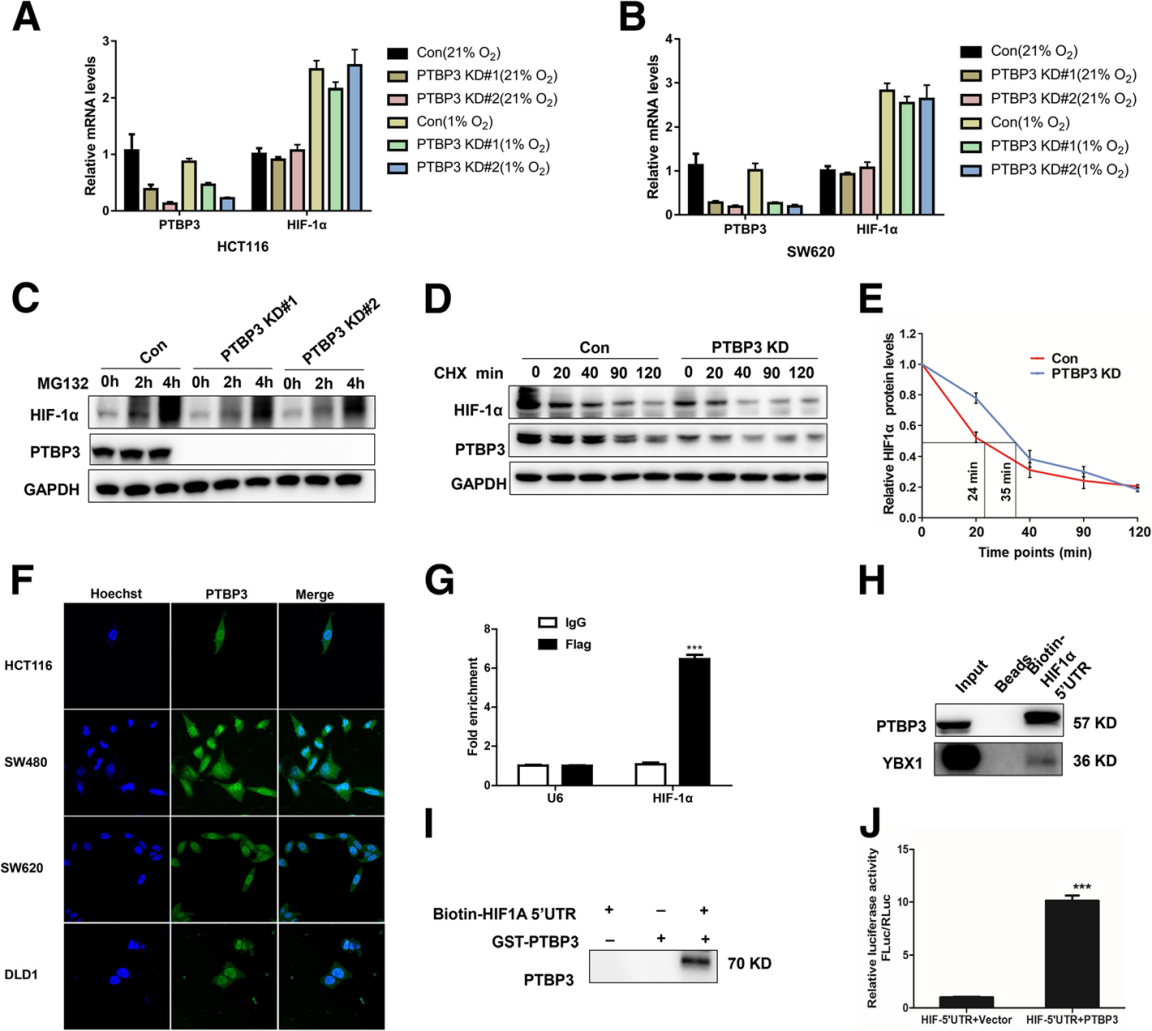
PTBP3 directly binds to HIF-1α mRNA to active its translation in colon cancer cells. **a** Relative HIF-1α mRNA expression measured by qRT-PCR in HCT116 ± PTBP3 KD under normoxia and hypoxia (1% O_2_) 4 h. Values were normalized against GAPDH from three independent experiments and are presented as means ± SD. **b** Relative HIF-1α mRNA expression measured by qRT-PCR in SW620 cells ± PTBP3 KD under normoxia and hypoxia (1% O_2_) 4 h. Values were normalized against GAPDH from three independent experiments and are presented as means ± SD. **c** Western blot showing effects of the MG132 proteasome inhibitor on HIF-1α protein accumulation at the indicated time points for the HCT116 cell line ± PTBP3 KD under 1% O_2_.GAPDH was used as a loading control. **d** Western blot showing HIF-1α protein decay in HCT116 cells ± PTBP3 KD at the indicated time points after cyclohexamide (CHX) addition under 1% O_2_. GAPDH was used a loading control. **e** Graphical representation of HIF1α protein levels from (**d**) based on densitometry. Half-lives are shown under the curves, representing results of two independent experiments ± SD. **f** PTBP3 intracellular localization was visualized in colon cancer cells: HCT116, SW480, SW620 and DLD1 by immunofluorescence assays. **g** The interaction of HIF-1α 5′UTR with PTBP3 was verified by an RNA immunoprecipitation (RIP) assay. Data are presented as the means ± SD for experiments in triplicate. **h** Western blot of PTBP3 and YBX1 in RNA pull-down samples by in vitro transcribed biotin labeled HIF-1α 5′UTR RNA in HCT116 cells. **i** Western blot of PTBP3 in RNA pull-down samples by in vitro transcribed biotin labeled HIF-1α 5′UTR RNA and PTBP3 GST fusion protein. **j** Relative HIF1a-5′UTR -Luc activity in HCT116 cells ± PTBP3 OE was detected by the Dual Luciferase Reporter Assay System. The Rluc activity was used for normalization. Data are presented as the means ± SD for experiments in triplicate. ***p < 0.001

We next investigated how PTBP3 regulates HIF-1α protein expression since the PTB members reportedly act both through regulating mRNA stability and translational mechanism [23, 24]. We first calculated the mRNA levels of HIF-1α in PTBP3 KD cells under normoxia or hypoxia. The HIF-1α mRNA stayed the same after PTBP3 KD as the control under normoxia or hypoxia (Fig. 5a-b). The data suggested that PTBP3 exhibited minimal effect on the stability of HIF-1α mRNA. We also assessed the potential effects of PTBP3 on HIF-1α protein stability. We treated the HCT116 cells with the MG132 proteasome inhibitor and failed to completely rescue HIF-1α protein levels under hypoxia in HCT116 with PTBP3 KD (Fig. 5c). Moreover, we used cycloheximide (CHX) to block translation and measured HIF1a degradation rates. The data showed that PTBP3 KD had little effect on the degradation rates of HIF-1α protein (Fig. 5d-e). These data presented that PTBP3 had little effects on HIF-1α mRNA and protein stability, and indicated PTBP3 may regulate HIF-1α protein level through activating HIF-1α translation.

To further verify the probable mechanism of PTBP3 in translational regulating HIF-1α, we firstly tested the cellular location of PTBP3 in CRC cells. The immunofluorescence assays showed that PTBP3 was located in both cytoplasm and nucleus (Fig. 5f), suggesting that PTBP3 may play a role both in cytoplasm and nucleus. We then accessed whether PTBP3 directly binds to HIF-1α mRNA and activates translation of HIF-1α. RNA immunoprecipitation (RIP) demonstrated that PTBP3 associates with HIF-1A transcripts (Fig. 5g). Next, we used biotin labelling HIF-1α 5′UTR mRNA to perform RNA pull-Down assay. This data showed that HIF-1α 5’UTR can not only contract with the endogenous PTBP3 but also directly bind to the GST-PTBP3 fusion protein (Fig. 5h-i). Further, we cloned the HIF-1α 5’UTR to the upstream of firefly LUC to access whether PTBP3 enhanced HIF-1α mRNA translation efficiency in cells. The dual-Luciferase reporter assay revealed significant LUC activity in PTBP3 OE cells versus the control (Fig. 5j). These data strongly supported a role for PTBP3 in translational activation of HIF-1α, likely through direct effects of PTBP3 on HIF-1α mRNA translational efficiency.

### YBX1 involves in PTBP3-mediated HIF-1α protein expression and malignant features in CRC

To explore the potential cofactors of PTBP3 in regulating HIF-1α translation, we identified the proteins that were associated with PTBP3 using Flag-tag pull-down followed by LC-MS (Additional file 4: Figure S2A). The data suggested that HNRNPM, UPF1, IGF2BP1 and YBX1 were associated with PTBP3. Next, we confirmed the interactions between PTBP3 with HNRNPM, UPF1, IGF2BP1 and YBX1 respectively using IP and Western blot (Additional file 4: Figure S2B). YBX1 was reported could interact with HIF-1α 5’UTR to promote HIF-1α translation in sarcoma [11], we speculated that YBX1 may play as a cofactor of PTBP3 in regulating HIF-1α translation. To verify the hypothesis, we first overexpressed YBX1 in HCT116 cells and tested HIF-1α protein level, whereas YBX1 OE failed to increase the expression of HIF-1α protein level (Additional file 4: Figure S2C). We silenced YBX1 in PTBP3 OE HCT116 cells using YBX1 siRNA, we noticed that YBX1 KD can partially decrease HIF-1α expression (Additional file 4: Figure S2D), as well as repressing cell migration and invasion induced by PTBP3 overexpression (Additional file 4: Figure S2E-G). These findings provided compelling evidence that PTBP3 and YBX1 may form a complex to regulate HIF-1α translation in colon cancer.

### HIF-1α is a critical effector of PTBP3-mediated malignant features in colon cancer

**Fig. 6:**
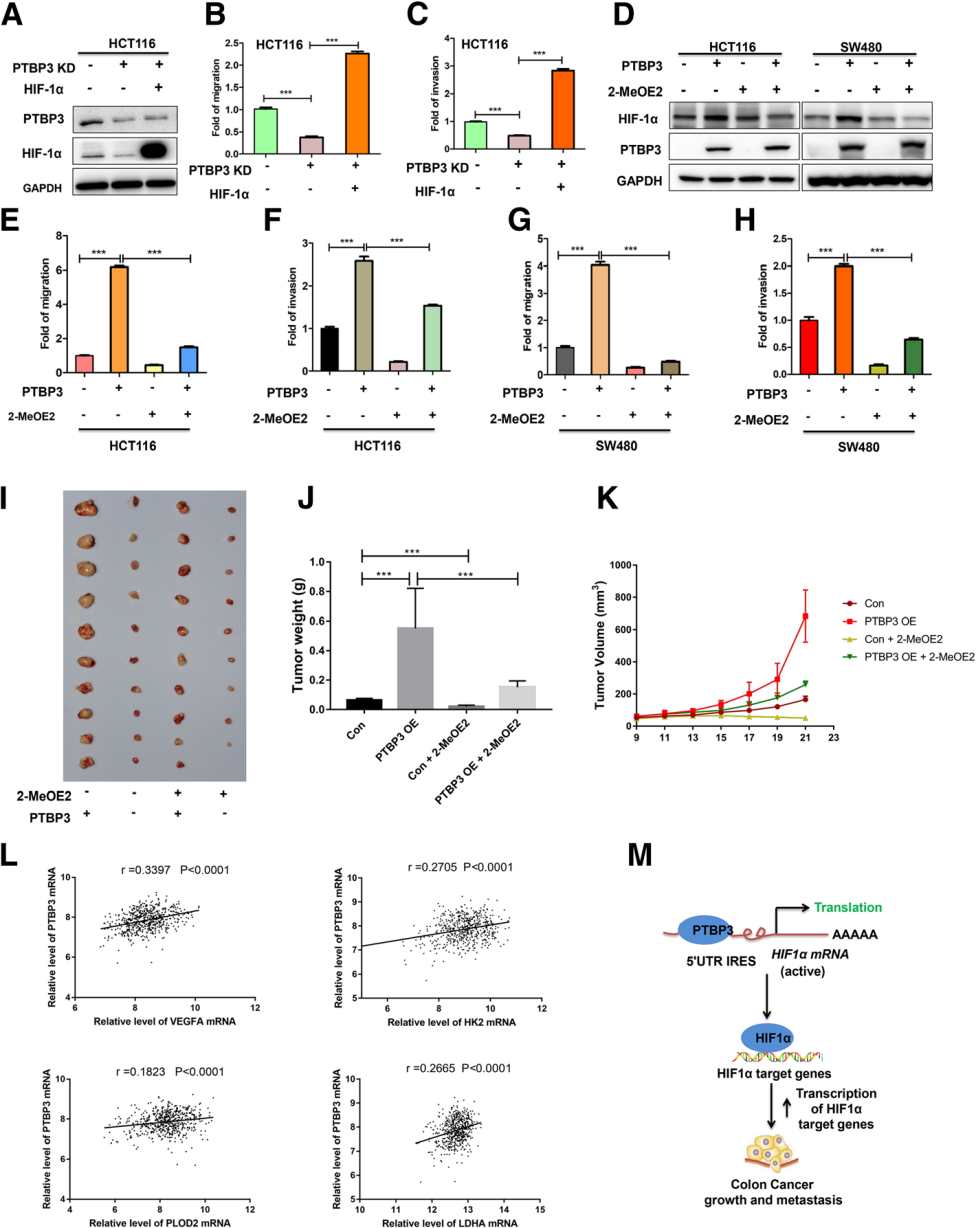
HIF-1α is a critical effector of PTBP3-mediated malignant features in colon cancer. **a** HCT116 cells ± PTBP3 KD were co-transfected with expression vectors encoding either empty vector, mutant HIF-1α and assessed by Western blot for HIF1α and PTBP3 expression. GAPDH was used as a loading control. **b** Effects of ectopic expression of mutant HIF-1α on migration capacity of HCT116 cells with PTBP3 KD. **c** Effects of ectopic expression of mutant HIF-1α on invasive capacity of HCT116 cells with PTBP3 KD. **d** HCT116 and SW480 cells cells ± PTBP3 OE was treated with HIF-1α inhibitor 2-Methoxyestradiol (2-MeOE2, 10 μM.) and assessed by Western blot for HIF1α and PTBP3 expression. GAPDH was used as a loading control. **e** Effects of 2-MeOE2 on migration capacity of HCT116 cells ± PTBP3 OE. **f** Effects of 2-MeOE2 on invasive capacity of HCT116 cells ± PTBP3 OE. **g** Effects of 2-MeOE2 on migration capacity of SW480 cells ± PTBP3 OE. **h** Effects of 2-MeOE2 on invasive capacity of SW480 cells ± PTBP3 OE. **i-k** Effect of 2-MeOE2 on the subcapsular tumor xenografts growth formed by injection of HCT116 cell ± PTBP3 OE, as assessed by evaluating tumor weight (**j**) and tumor volume (**k**). 2-MeOE2 was used at a dose of 50 mg/kg per day by means of intragastric administration for one week. **l** Correlation analysis of relative mRNA levels of PTBP3 and VEGFA, HK2, PLOD2 or LDHA using the GEO dataset (GSE40976), contains 566 CRC tissues. **m** A cartoon summarizing our findings. PTBP3 promotes HIF-1α protein expression through binding to the IRES sequences of the HIF-1α mRNA 5’UTR to enhance HIF-1α translation in CRC, thereby promoting colon cancer growth, and metastasis. Data are presented as the means ± SD for experiments in triplicate. ***p < 0.001

We assessed whether HIF-1α is important for PTBP3 induced migration and invasion of CRC cells. Firstly, we expressed mutant HIF-1α (resistant to HIF-1α degradation) in HCT116 PTBP3 KD cells (Fig. 6a). Re-expression of HIF-1α completely rescued the migration and invasion capacity of PTBP3 KD cells (Fig. 6b-c). Moreover, we used 2-MeOE2 (10 μM), which is a HIF-1α inhibitor that can inhibit HIF-1α activity [25], to block the HIF-1α pathway in HCT116 and SW480 PTBP3 OE cells to assess the importance of HIF-1α in PTBP3 induced malignant features. Data showed that 2-MeOE2 can partially reduce the expression of HIF-1α protein levels in SW480, but exhibited minimal effect on HIF-1α protein expression in HCT116 and cells (Fig. 6d). The results were consistent with the previous reports that 2-MeOE2 may not alter HIF-1α expression level in some kinds of cell types [25–27]. The blocking of HIF-1α pathway by 2-MeOE2 significantly repressed the migration and invasion abilities of PTBP3 OE cells (Fig. 6e-h). To assess the effect of HIF-1α on PTBP3-mediated tumor growth in vivo, we used 2-MeOE2 to block HIF-1α pathway in the xenografts formed by HCT116 PTBP3 Con/OE cells. Data showed that blocking the HIF-1α pathway by 2-MeOE2 dramatically reduced tumor volume and weight formed by HCT116 PTBP3 OE cells (Fig. 6i-k), highlighting HIF-1α as a critical effector of PTBP3-mediated malignant features. VEGFA, LDHA, HK2 and PLOD2 are the known target genes regulated by HIF-1α [21, 28–30]. Herein, we analyzed the correlation between PTBP3 mRNA level and HIF-1α target genes in public dataset (GEO: GSE40967), results showed that PTBP3 was positively correlated with VEGFA, LDHA, HK2 and PLOD2 (Fig. 6l). Our results suggested that PTBP3 mediated CRC malignant features through HIF-1α pathway.

In conclusion, our study reveals an important post-transcriptional control mechanism of the tumor progression master regulator HIF-1α. Our results suggested a model that PTBP3 promoted HIF-1α protein expression through binding to the IRES sequences of the HIF-1α mRNA 5’UTR to enhance HIF-1α translation in CRC, thereby promoting CRC angiogenesis, growth, and metastasis (Fig. 6m).

## Discussion

PTBP3, a paralog of PTBP1, contains more than 70% amino acid sequence similarities [4]. PTBP1 is a well-known RBP that regulates RNA alternative splicing (AS), decay, location, stability and translation. PTBP1 is known to play oncogenic functions in some kinds of cancers, including gliomas, breast, bladder, pancreatic and colon cancers [31–35]. By contrast, the molecular functions or physiological roles of PTBP3 have been relatively neglected. Recently, we and others have showed that PTBP3 was overexpressed and functioned as an oncogene in breast, gastric and hepatocellular cancers [5, 6, 36]. PTBP3 may present an emerging role of in various cancer progressions. In this study, we systematically investigated the roles of PTBP3 and provided evidence of potential molecular mechanism in CRC. Our results showed that PTBP3 expression was positively correlated with high TNM stage and poor five-year survival of CRC patients using TMA. In addition, we have demonstrated that PTBP3 promoted tumor growth and metastasis via enhancing the translation of HIF-1α, as proven by a series of in vitro and in vivo experiments. Therefore, PTBP3 plays critical role in CRC.

One of the major findings of our study was that the protein level of PTBP3 was significantly higher in CRC tissues compared with the adjacent normal colon tissues from CRC patients, suggesting a biomarker role of PTBP3 in CRC. Multivariate Cox’s regression analysis indicated that high PTBP3 expression was an independent prognostic factor for OS and DFS. High PTBP3 expression was also correlated with high TNM stage and depth of invasion of CRC. These results suggested that PTBP3 expression can be used as a prognostic biomarker in CRC. PTBP3 was overexpressed in breast, gastric and hepatocellular cancers [5, 6, 36], suggesting PTBP3 might widely express in different kinds of cancers and be a potential biomarker for tumors.

To further understand the biological function of PTBP3 in CRC processions, we investigated the malignant features of PTBP3 in colon cancer cell lines, HCT116, SW480 and SW620 using PTBP3 KD or OE lentivirus infection. Previous studies highlighted the oncogenic role of PTBP3 in tumor migration, invasion, growth and metastasis [5, 36]. Consistent with these previous data, our results showed that PTBP3 KD and OE reduced tumor cell growth, migration, invasion abilities, respectively. Our data also showed that PTBP3 promoted tumor growth and lung metastasis in an animal model of CRC. We also showed that PTBP3 also contributed the angiogenesis ability of colon cancer cells in vitro and in vivo. These results clearly emphasised the importance of PTBP3 in CRC procession.

Another major finding of our study was the clarifying of the molecular mechanism of PTBP3 in regulating CRC procession. HIF-1α has been recognized as an important drug target in kinds of cancers. Strong correlation between elevated levels of HIF-1α and tumor metastasis, angiogenesis and poor patient prognosis, as well as tumor resistance therapy, has been proven [37–39]. HIF-1α can be regulated by NF­κB at the transcription level [40–42]. Moreover, mTORC1 and mTORC2 can promote HIF-1α protein expression by activating HIF-1α mRNA translation [43]. An RNA binding protein, YBX1, can also promote HIF-1α mRNA translation depending on binding to the IRES sequence of 5′UTR of HIF-1α mRNA [11]. In this work, our data showed PTBP3 KD and OE exhibited minimal effect on HIF-1α mRNA expression, but significantly regulated the protein level of HIF-1α in CRC. Results also showed PTBP3 did not regulate HIF-1α protein stability, considering that PTBP3 is a paralog of PTBP1, and PTBP1, which could promote mRNA translation through binding to IRES sequence [44]. We speculated that PTBP3 may regulate HIF-1α protein expression by regulating HIF-1α mRNA translation. Our RNA pull-down assay revealed that PTBP3 can directly interact with the IRES sequence of 5’UTR of HIF-1α, and the luciferase report assay indicated that PTBP3 could enhance HIF-1α mRNA translation by binding to the IRES region of HIF-1α mRNA. We also revealed that the blocking of HIF-1α pathway using HIF-1α inhibitor 2-MEOE2 can significantly reduce PTBP3 induced colon cancer cell migration and invasion in cultured cell lines, as well as tumor growth in animal xenografts model. These results suggested that developing new inhibitors that specifically target PTBP3 may offer a novel therapeutic approach to block CRC growth and metastasis.

## Conclusions

In summary, this study provides novel evidence for the clinical and biological significance of PTBP3 in CRC. Our data demonstrated that PTBP3 was highly expressed in CRC patients and was positively correlated with poor five-year survival of CRC patients. Importantly, we discovered a new molecular mechanism of PTBP3 in activating HIF-1α translation in CRC procession. Our data showed that PTBP3 was an important regulator of HIF-1α and promoted CRC tumorigenesis and metastasis procession. We conclude that quantification of PTBP3 expression may serve as a clinically diagnostic and prognostic biomarker and potentially as a therapeutic target in CRC.

## Additional files

Additional file 1: **Table S1.** Univariate Cox regression analysis of PTBP3 expression and clinicopathologic variables predicting the survival of CRC patients. (DOCX 16 kb)

Additional file 2: **Table S2.** Multvariate Cox regression analysis of PTBP3 expression on 5-year overall and disease specific survival of CRC patients. (DOCX 15 kb)

Additional file 3: **Figure S1.** Kaplan–Meier survival curves depicting overall survival stratified by PTBP3 protein expression levels in different histologic subtypes for CRC. (A) Kaplan–Meier survival curves depicting overall survival stratified by PTBP3 protein expression levels in histology grade I and II. (B) Kaplan–Meier survival curves depicting overall survival stratified by PTBP3 protein expression levels in histology grade III and IV (*p* < 0.001). (C) Kaplan–Meier survival curves depicting overall survival stratified by PTBP3 protein expression levels in stage pT1/T2 (*p* < 0.001). (D) Kaplan–Meier survival curves depicting overall survival stratified by PTBP3 protein expression levels in stage pT3/T4 (*p* < 0.001). (E) Kaplan–Meier survival curves depicting overall survival stratified by PTBP3 protein expression levels in stage pN0 (*p* < 0.001). (F) Kaplan–Meier survival curves depicting overall survival stratified by PTBP3 protein expression levels in stage pN1/N2/N3 (*p* < 0.001). (TIF 564 kb)

Additional file 4: **Figure S2.** Effect of YBX1 on PTBP3-mediated malignant features in CRC. (A) Proteins that were associated with PTBP3 using Flag-tag pull-down followed by LC-MS. (B) Detection of the interactions between PTBP3 with HNRNPM, UPF1, IGF2BP1 and YBX1 respectively using IP and Western blot assays. (C) Effect of YBX1 OE on the expression of HIF-1α protein level, as assessed by Western blot. (D) Effect of YBX1 KD on the expression of HIF-1α protein level in HCT116 cell ± PTBP3 OE, as assessed by Western blot. (E) Cell migration and invasion of HCT116 cells ± PTBP3 OE. (F) Relative migration fold changes in HCT116 cells ± PTBP3 OE. (G) Relative migration fold changes in HCT116 cells ± PTBP3 OE. Data are presented as the means ± SD for experiments in triplicate. ***p < 0.001. (TIF 1181 kb)

## Data Availability

The datasets used and/or analysed during the current study are available from the corresponding author on reasonable request.

## Abbreviations

2-MeOE2: 2-Methoxyestradiol
AS: Alternative splicing
CHX: Cycloheximide
CRC: Colorectal cancer
DFS: Disease-free survivals
HIF-1α: Hypoxia-inducible transcription factors 1α
IRES: Internal ribosome entry site
OS: Overall survival
PTBP3: Polypyrimidine tract-binding protein 3
RBPs: RNA-binding proteins
RIP: RNA immunoprecipitation
TMA: Tissue microarray
VEGF: Vascular endothelial growth factor
