# Supplementary material for: PTBP3 contributes to colorectal cancer growth and metastasis via translational activation of HIF-1α
Source: J Exp Clin Cancer Res. 2019 Jul 10;38:301. doi: 10.1186/s13046-019-1312-y (PMC6622005; doi:10.1186/s13046-019-1312-y)
Supplement: Supplementary file 1 — Table S1. Univariate Cox regression analysis of PTBP3 expression and clinicopathologic variables predicting the survival of CRC patients. (DOCX 16 kb) [file 13046_2019_1312_MOESM1_ESM.docx]

**Supplementary Table S1.** Univariate Cox regression analysis of PTBP3 expression and clinicopathologic variables predicting the survival of CRC patients

| Variables† | Overall survival | |  | Disease-specific survival | | |
| --- | --- | --- | --- | --- | --- | --- |
|  | HR (95%CI) | *P* |  | HR (95%CI) |  | *P* |
| PTBP3 | 0.276 (0.198–0.385) | <0.001 |  | 0.154 (0.089–0.266) |  | <0.001 |
| Age | 0.663 (0.471–0.933) | 0.019 |  | 0.693(0.414–1.160) |  | 0.163 |
| Gender | 0.778 (0.562–1.078) | 0.131 |  | 0.732 (0.446–1.200) |  | 0.216 |
| Distant metastasis | 0.379 (0.192–0.764) | 0.005 |  | 0.260 (0.104–0.653) |  | 0.004 |
| TNM stage | 0.139 (0.092–0.209) | <0.001 |  | 0.096 (0.049–0.191) |  | <0.001 |
| Differentiate | 1.514 (1.023–2.241) | 0.038 |  | 2.593 (1.498–4.490) |  | 0.001 |
| Tumor diameter | 0.777 (0.561–1.074) | 0.127 |  | 0.785 (0.478–1.293) |  | 0.240 |
| Depth of invasion | 0.557 (0.350–0.884) | 0.013 |  | 0.267 (0.107–0.667) |  | 0.005 |

Abbreviations: HR: hazard ratio; CI: confidence interval; LNM: lymph node metastasis

†: PTBP3: low *vs* high; Age: ≤60 *vs* >60; Gender: male *vs* female; Depth of invasion: T1-T2 *vs* T3-T4; Distant metastasis: M0 *vs* M1; Differentiate: poor *vs* moderate and high; TNM stage was ranked as I-II *vs* III- IV; Tumor diameter: <5 *vs* ≥5.
