# Supplementary material for: PTBP3 contributes to colorectal cancer growth and metastasis via translational activation of HIF-1α
Source: J Exp Clin Cancer Res. 2019 Jul 10;38:301. doi: 10.1186/s13046-019-1312-y (PMC6622005; doi:10.1186/s13046-019-1312-y)
Supplement: Supplementary file 2 — Table S2. Multvariate Cox regression analysis of PTBP3 expression on 5-year overall and disease specific survival of CRC patients. (DOCX 15 kb) [file 13046_2019_1312_MOESM2_ESM.docx]

**Supplementary Table S2.** Multvariate Cox regression analysis of PTBP3 expression on 5-year overall and disease specific survival of CRC patients

| **Variable †** | **Overall survival** | | | **Disease-specific survival** | | |
| --- | --- | --- | --- | --- | --- | --- |
|  | **Hazard ratio** | **95％CI ‡** | **P*** | **Hazard ratio** | **95％CI^†^** | **P*** |
| PTBP3 | 2.484 | 1.761-3.502 | <0.001 | 4.830 | 2.763-8.445 | <0.001 |
| Age | 1.363 | 0.959-1.937 | 0.084 | 1.431 | 0.838-2.442 | 0.189 |
| Gender | 1.345 | 0.961-1.884 | 0.084 | 1.461 | 0.877-2.432 | 0.126 |
| Tumor size | 1.354 | 0.969-1.892 | 0.076 | 1.274 | 0.775-2.147 | 0.364 |
| TNM stage | 5.962 | 3.894-9.127 | <0.001 | 7.733 | 3.902-15.324 | <0.001 |
| Distant metastasis | 1.996 | 0.998-3.991 | 0.051 | 2.345 | 0.909-6.047 | 0.078 |

†Coding of variables: Cancer was coded as 1 (negative), and 2 (positive). Age was coded as 1 (≤ 60 years), and 2 (> 60` years). Gender was coded as 1 (male), and 2 (female).Tumor size was coded as 1 (<5cm), and 2 (≥5 cm). TNM stage was coded as 1 (I-II), and 2 (III-IV). Distant metastasis was coded as 1 (M0), and 2 (M1).

‡CI: Confidence interval.
