# Supplementary figures and images for: PTBP3 contributes to colorectal cancer growth and metastasis via translational activation of HIF-1α
Source: J Exp Clin Cancer Res. 2019 Jul 10;38:301. doi: 10.1186/s13046-019-1312-y (PMC6622005; doi:10.1186/s13046-019-1312-y)

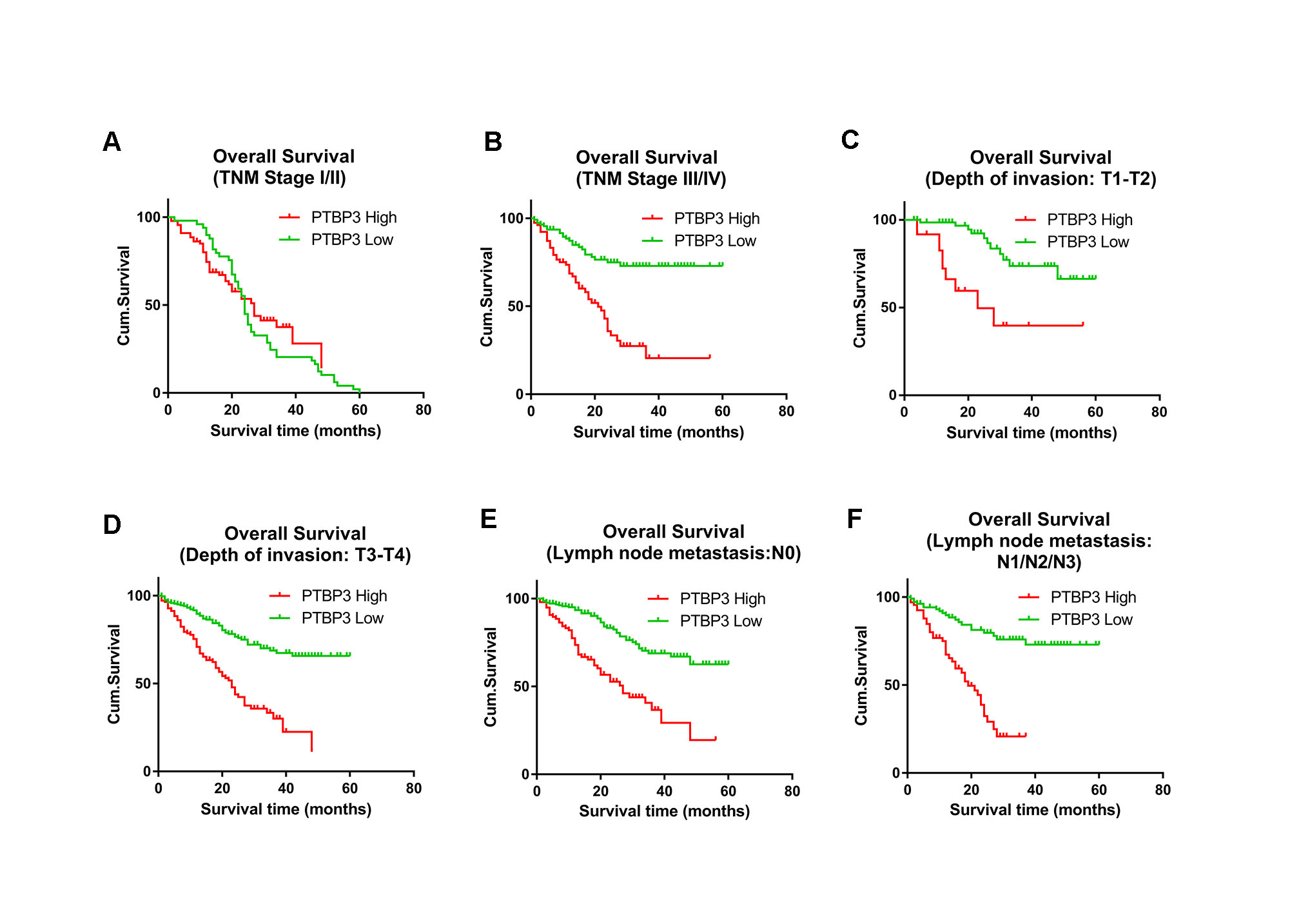

Supplement: Supplementary file 3 — Figure S1. Kaplan–Meier survival curves depicting overall survival stratified by PTBP3 protein expression levels in different histologic subtypes for CRC. (A) Kaplan–Meier survival curves depicting overall survival stratified by PTBP3 protein expression levels in histology grade I and II. (B) Kaplan–Meier survival curves depicting overall survival stratified by PTBP3 protein expression levels in histology grade III and IV (p < 0.001). (C) Kaplan–Meier survival curves depicting overall survival stratified by PTBP3 protein expression levels in stage pT1/T2 (p < 0.001). (D) Kaplan–Meier survival curves depicting overall survival stratified by PTBP3 protein expression levels in stage pT3/T4 (p < 0.001). (E) Kaplan–Meier survival curves depicting overall survival stratified by PTBP3 protein expression levels in stage pN0 (p < 0.001). (F) Kaplan–Meier survival curves depicting overall survival stratified by PTBP3 protein expression levels in stage pN1/N2/N3 (p < 0.001). (TIF 564 kb) [file 13046_2019_1312_MOESM3_ESM.tif]

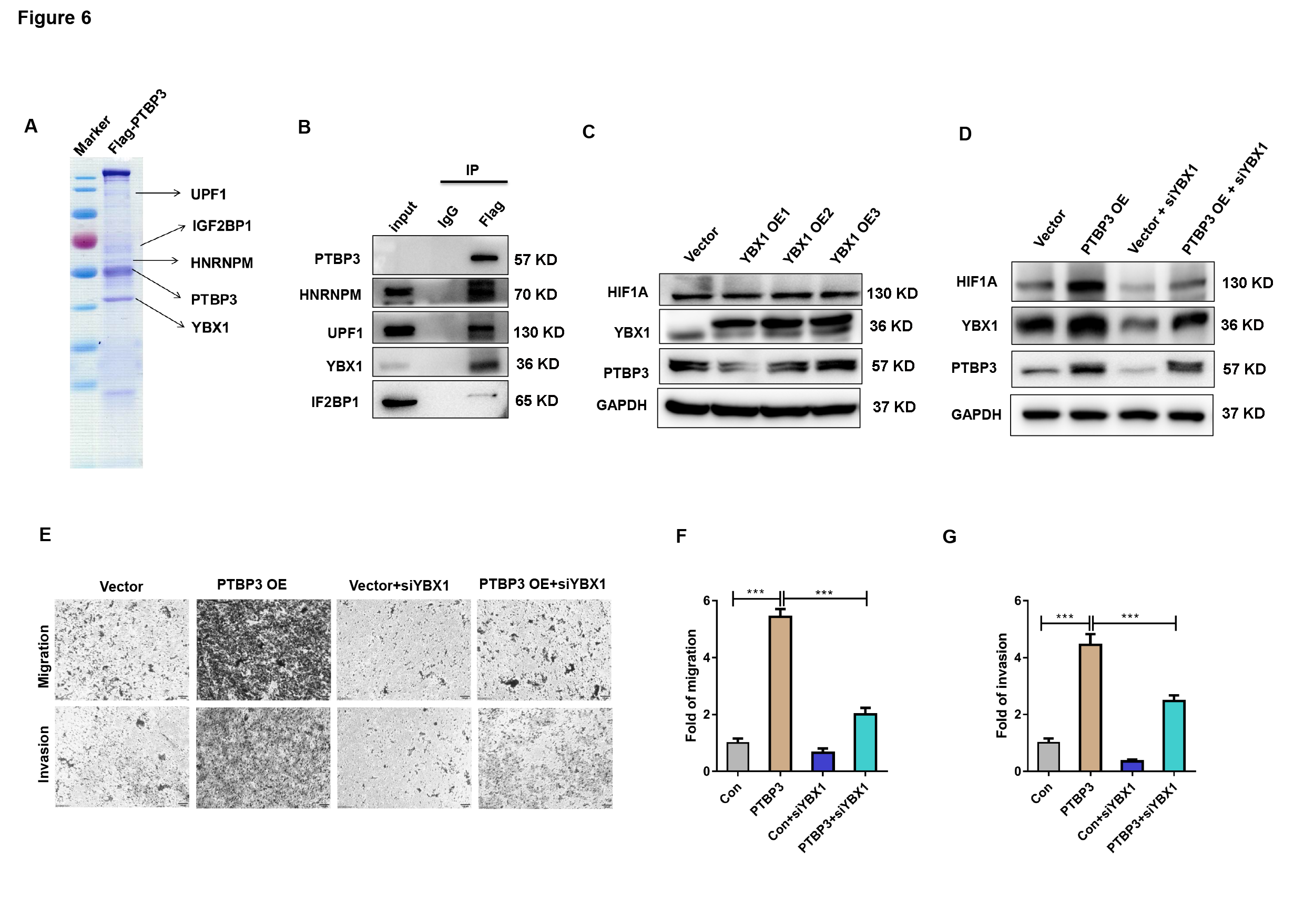

Supplement: Supplementary file 4 — Figure S2. Effect of YBX1 on PTBP3-mediated malignant features in CRC. (A) Proteins that were associated with PTBP3 using Flag-tag pull-down followed by LC-MS. (B) Detection of the interactions between PTBP3 with HNRNPM, UPF1, IGF2BP1 and YBX1 respectively using IP and Western blot assays. (C) Effect of YBX1 OE on the expression of HIF-1α protein level, as assessed by Western blot. (D) Effect of YBX1 KD on the expression of HIF-1α protein level in HCT116 cell ± PTBP3 OE, as assessed by Western blot. (E) Cell migration and invasion of HCT116 cells ± PTBP3 OE. (F) Relative migration fold changes in HCT116 cells ± PTBP3 OE. (G) Relative migration fold changes in HCT116 cells ± PTBP3 OE. Data are presented as the means ± SD for experiments in triplicate. ***p < 0.001. (TIF 1181 kb) [file 13046_2019_1312_MOESM4_ESM.tif]
